# Internal urethrotomy versus Optilume™ drug-coated balloon dilatation for the management of anterior urethral strictures: morbidity comparison of the techniques

**DOI:** 10.1007/s00345-026-06596-7

**Published:** 2026-07-07

**Authors:** Alexander Güdemann, Giovanni Lorenzo De Giorgi, Leonidas Karapanos, Philipp Kovacs, Guglielmo Mantica, Lea Seiler, Johannes Engesser, Pascal Viktorin, Svetozar Subotic, Andreas Sauer, Stephen Wyler, Maciej Kwiatkowski, Luca Afferi

**Affiliations:** 1 Department of Urology, Kantonsspital Aarau, Aarau, Switzerland; 2 Department of Urology, Kantonsspital Baselland, Liestal, Switzerland; 3 Department of Urology, Klinikum Leverkusen, Leverkusen, Germany; 4 Department of Urology, IRCCS Ospedale Policlinico San Martino, Genova, Italy; 5 Department of Urology, Luzerner Kantonsspital, Lucerne, Switzerland; 6 Medical Faculty, University of Basel, Basel, Switzerland; 7 Department of Urology, Academic Hospital Braunschweig, Braunschweig, Germany

**Keywords:** Urethral stricture, Optilume, Internal urethrotomy, Lower urinary tract

## Abstract

**Introduction:**

Direct visual internal urethrotomy (DVIU) for short anterior urethral strictures is associated with a high rate of stricture recurrence. Optilume™ drug-coated balloon (ODCB) has recently emerged as a minimally invasive treatment alternative. However, comparative data between ODCB and DVIU are limited. This multicenter study aims to compare the two procedures in terms of functional outcomes and stricture-free survival (SFS) in patients with anterior urethral strictures.

**Methods:**

This multicenter retrospective study included 140 patients treated for anterior urethral stricture under 3 cm in length with either ODCB dilatation or internal urethrotomy. Data were collected from five European tertiary referral centers between November 2010 and April 2026. Stricture length was assessed by either cystoscopy, retrograde urethrography, or both. Stricture recurrence during follow-up was defined as symptomatic restenosis requiring reintervention. Multivariable Cox regression analysis was performed to identify predictors of recurrence. SFS was estimated using Kaplan-Meier estimates, with group comparisons assessed using the log-rank test.

**Key findings:**

A total of 68 (48%) patients underwent DVIU and 72 (52%) ODCB. Prior stricture treatment had been performed in 6 (9%) in the internal urethrotomy group and 56 (78%) in the ODCB group (*p* < 0.001). Prior dilatations were performed in 6 (9%) patients for DVIU and 27 (38%) for ODCB dilatation (*p* < 0.001). The groups were otherwise similar with regards to baseline characteristics, including stricture length, diabetes mellitus, stricture site. The median follow-up was 12 months (IQR: 4–29) in the internal urethrotomy group and 9.5 months (IQR: 5–14) in the ODCB group. Postoperative complications were reported in 3% of patients after DVIU and 6% after ODCB dilatation. Median preoperative IPSS was 20.5 (15–26) for DVIU and 18.5 (11–25) for ODCB dilatation (*p* = 0.4). The median IPSS after three months was 16 (13–20) for DVIU and 6.5 (3–11) for ODCB dilatation (*p* < 0.001). The type of surgery was not predictive of stricture recurrence at multivariable cox regression analysis. SFS after 12 months was 67% (95% CI: 56–80%) for internal urethrotomy and 68% (95% CI: 50–82%) for Optilume™ (*p* = 0.81).

**Conclusions:**

Despite being used in a substantially more pretreated cohort, ODCB dilatation showed similar safety and SFS after 12 months as compared to DVIU, suggesting a potential role as an effective salvage strategy after failed endoscopic treatment or even early line treatment. Future prospective studies are needed to better define selection criteria and optimal treatment sequencing.

## Introduction

Anterior urethral strictures are characterized by a narrowing of the anterior urethra consisting of fibrotic tissue, leading to lower urinary tract symptoms, recurrent urinary tract infections and reduction in quality of life [1, 2]. For short anterior strictures, which are commonly defined as strictures under 2 cm in length, direct visual internal urethrotomy (DVIU) is one of the most commonly used procedures due to its technical simplicity, low perioperative morbidity and short recovery time [1, 3]. Long-term outcomes, however, show a high recurrence [2, 4–6]. 

In recent years, Optilume™ drug-coated balloon (ODCB) has become an alternative for DVIU [1, 5, 7–10]. The procedure includes a dilatation of the urethral stricture combined with a topical delivery of Paclitaxel, an antiproliferant agent that inhibits fibroblast activity, into the scar tissue, aiming to reduce recurrence of scar tissue formation [7, 8, 11–13]. Early prospective studies, including the ROBUST trial series, have shown promising short- and mid-term outcomes in terms of functional outcomes, patient safety and quality of life [7, 8]. These findings have led to a growing adoption of ODCB in the treatment of short anterior urethral stricture [1, 3, 7, 8]. 

Current evidence directly comparing ODCB dilatation and DVIU is limited. The primary objective of this study was to conduct a comparative analysis of ODCB dilatation with DVIU, focusing on functional outcomes and stricture-free survival (SFS). By evaluating these two endoscopic approaches, this study seeks to clarify whether ODCB offers a meaningful advantage over DVIU and to better define its role in the management algorithm of anterior urethral strictures [1, 3]. 

## Methods

### Study population

This is a retrospective analysis of a multicenter cohort of patients undergoing either ODCB dilatation or DVIU between November 2010 and April 2026 at five European referral centers. The study was approved by the Ethics Committee Northwest and Central Switzerland (EKNZ; BASEC Nr. 2025 − 01438). No age limits were applied.

Demographic data included age at surgery, smoking history, diabetes mellitus, site of stricture, length of stricture, prior dilatations, previous surgeries of the urethra, International Prostate Symptom Score (IPSS). Intraoperative data included intraoperative complications, type of intervention, postoperative complications, length of indwelling catheter. Postoperative data included 3-month IPSS, peak urinary flow, post-void volume and persisting stricture during follow-up. Postoperative complications were classified according to the Clavien-Dindo Classification (CDC) and intended as any complication within 30 days of surgery.

## Eligibility criteria

Main inclusion criteria were patients with a stricture of the anterior urethra, either primary or after any previous stricture treatment. Patients underwent a follow-up of at least 3 months after surgical treatment for the urethral stricture. Exclusion criteria included a history of urethral malignancy, prior radical prostatectomy, radiation of the pelvis and the presence of lichen sclerosus. Patients with a penile prosthesis or an artificial urinary sphincter were also excluded, as well as those who had received intradetrusor botulinum toxin-A injections within the previous 12 months.

## Surgical procedures

DVIU is performed under general or regional anesthesia with the patient in the lithotomy position. After localization of the urethral stricture via rigid urethroscopy, a guide wire is placed through the stricture into the bladder. An incision is made at the 12 o’clock position using the urethrotome, until the lumen is opened adequately and healthy mucosa is reached. Passage of the instrument into the bladder confirms sufficient incision of the stricture. Afterwards, a foley catheter is placed and left indwelling for up to three days [1]. 

ODCB is also performed under general or regional anesthesia with the patient in the lithotomy position. After cystoscopic localization of the stricture, a guide wire is placed through the stricture into the bladder. Optional dilation may be performed using either a single-use catheter or a non-coated dilation balloon (24 Fr), depending on the location and length of the stricture.

Subsequently, the Optilume™ paclitaxel-coated balloon catheter (30 Fr) is positioned across the stricture under fluoroscopic or endoscopic guidance, ensuring that the balloon length extended at least 5 mm beyond both ends of the stricture. The balloon is inflated for a duration of five minutes and a pressure of 10 atm [8]. After deflation and removal of the balloon, correct urethral patency is confirmed endoscopically. Afterwards, a foley catheter is placed for one to three days [7, 8]. 

## Outcomes

The main outcome of this study was SFS after treatment with Optilume versus DVIU. SFS was defined as the time from urethral stricture treatment to symptomatic stricture relapse. Any symptomatic stricture requiring any sort of treatment was confirmed and described by urethrocystoscopy or retrograde urethrogram.

### Statistical analysis

Categorical variables were summarized as counts and percentages. Continuous variables were expressed as means with standard deviations (SD) or as medians with interquartile ranges (IQR). The recurrence of strictures was reported as the number and proportion of patients who experienced a recurrence. A multivariable cox-regression analysis to identify predictors of stricture recurrence was performed. Kaplan-Meier estimates were used to display SFS during follow-up. The log-rank test was used to describe statistically significant differences between the two curves. A two-sided p-value < 0.05 was used to identify statistically significant differences. All statistical analyses were performed using R (R Project, Vienna, Austria).

## Results

### Patients baseline characteristics

Patients baseline characteristics are described in Table 1. Median age at surgery was 61 (IQR: 44–72) for DVIU and 61 (IQR: 46–72) for Optilume DCB (*p* = 0.7). Baseline characteristics between the two groups were similar in regard to diabetes mellitus (*p* = 0.7), smoking history (*p* = 0.6) and type of stricture (*p* = 0.9). Previous stricture treatment was recorded in 56 (78%) patients undergoing Optilume DCB dilatation and in 6 (9%) patients undergoing DVIU (*p* < 0.001). Prior dilatations were performed in 27 (38%) patients undergoing ODCB and in 6 (9%) patients undergoing DVIU (*p* < 0.001). Median stricture length in DVIU was 1 cm (IQR: 0.5-1) versus 1.5 cm (IQR: 1–3) in Optilume (*p* < 0.001).

**Table 1:** Baseline characteristics of 140 patients treated with internal urethrotomy or Optilume for anterior urethral strictures

	Overall (*N* = 140, 100%)	Internal urethrotomy (*N* = 68, 48%)	Optilume (*N* = 72, 52%)	*P*-value
Age at surgery [years], median (IQR)	61 (44–72)	61 (44–72)	61 (46–72)	0.7
Diabetes mellitus, n (%)	22 (16)	10 (15)	12 (17)	0.7
Smoking history, n (%)				
No	114 (81)	56 (82)	58 (81)	0.6
Ex smoker	17 (12)	6 (9)	11 (15)	
Active smoker	9 (6)	6 (9)	3 (4)	
Type of stricture, n (%)				
Idiopathic	59 (42)	27 (40)	32 (44)	0.9
Iatrogenic	68 (49)	35 (52)	33 (46)	
Post-traumatic	8 (6)	3 (4)	5 (7)	
Inflammatory	5 (4)	3 (4)	2 (3)	
Previous stricture treatment, n (%)	62 (44)	6 (9)	56 (78)	< 0.001
Type of previous treatment, n (%)				
No previous treatment	78 (56)	62 (91)	16 (22)	
Only dilatations	21 (15)	6 (9)	15 (21)	
Internal urethrotomy +/- dilatations	19 (14)	0 (0)	19 (26)	
End-to-end urethroplasty	1 (1)	0 (0)	1 (1)	
Buccal mucosal urethroplasty	21 (15)	0 (0)	21 (30)	
Prior dilatations, n (%)	33 (24)	6 (9)	27 (38)	< 0.001
Number of prior dilations, median (IQR)	2 (1–5)	1 (1–2.5)	2 (1–5.5)	0.2
Stricture length [cm], median (IQR)	1 (1–2)	1 (0.5–1)	1.5 (1–3)	< 0.001

## Intra- and postoperative characteristics

Intra- and postoperative characteristics are described in Table 2. Postoperative complications were reported in 2 (3%) cases for DVIU and 4 (6%) cases for Optilume (*p* = 0.8). Days of indwelling catheter were comparable for DVIU (*n* = 2, IQR: 2–2) and Optilume (*n* = 1.5, IQR: 0–2) (*p* = 0.4). The median follow-up was 12 (IQR 4–28) months for DVIU and 9.5 (IQR: 5–14) for ODCB (*p* < 0.001). Persisting strictures during follow-up occurred in 43 (63%) cases for DVIU and 21 (29%) cases for ODCB.

**Table 2:** Intra- and postoperative characteristics of 140 patients treated with internal urethrotomy or Optilume for anterior urethral strictures

	Internal urethrotomy (*N* = 68, 48%)	Optilume (*N* = 72, 52%)	*P*-value
Postoperative complications*, *n* (%)	2 (3)	4 (6)	0.8
Days of indwelling urethral catheter, median (IQR)	2 (2–2)	1.5 (0–2)	0.4
Months of follow-up, median (IQR)	12 (4–28)	9.5 (5–14)	***< 0.001***
Persisting stricture during follow-up, n (%)	43 (63)	21 (29)	***< 0.001***

* within 30 days from surgery

### Preoperative and postoperative functional characteristics

Preoperative and postoperative functional characteristics are described in Table 3. Preoperative baseline characteristics were similar regarding peak urinary flow (*p* = 0.8), post-void residual (*p* = 0.5) and median IPSS (*p* = 0.3). Median peak urinary flow after 3 months for DVIU was 13 ml/s (IQR: 7–23) in comparison to 18 ml/s for Optilume (IQR: 14–23). Post-void residual volume at 3 months was 10 ml for DVIU (IQR: 0–50) and 10 ml for Optilume (IQR: 0–40). IPSS after 3 months was 6.5 (IQR: 3–11) in the Optilume group in comparison to 16 (IQR: 13–20) in the DVIU group (*p* < 0.001).

**Table 3:** Preoperative and postoperative functional characteristics of 140 patients treated with internal urethrotomy or Optilume for anterior urethral strictures. Variables are expressed as median and interquartile range

Variable	Internal urethrotomy (*N* = 68, 48%)	Optilume (*N* = 72, 52%)	*P*-value
Preoperative functional characteristics			
Peak urinary flow (Qmax) before surgery [ml/s]	6.1 (4.6–9.2)	6.6 (3.4–9.3)	0.8
Post-void residual before surgery [ml]	40 (0–110)	40 (0–150)	0.5
IPSS before surgery, median (IQR)	20 (15–26)	18 (11–25)	0.3
Postoperative functional characteristics			
Peak urinary flow (Qmax) at 3 months [ml/s]	13 (7.2–23)	18 (14–23)	0.2
Post-void residual at 3 months [ml]	10 (0–50)	10 (0–40)	0.1
IPSS at 3 months, median (IQR)	16 (13–20)	6.5 (3.0–11)	***< 0.001***

### Factors predictive of stricture recurrence

Multivariable Cox regression analysis evaluating factors predictive of stricture at follow-up is reported in Table 4. There was no factor predicting stricture recurrence at follow-up. Factors included were age, diabetes mellitus, type of stricture, recurrent stricture vs. previously untreated stricture and type of urethral surgery.

**Table 4:** Multivariable cox regression analysis evaluating factors predictive of stricture recurrence at follow-up

Variable	HR (95%CI)	*P*-value
Age (years)	1.01 (0.99, 1.03)	0.3
Diabetes mellitus	1.24 (0.76, 2.03)	0.4
Type of stricture		
Idiopathic	*Ref.*	*Ref.*
Iatrogenic	1.19 (0.64, 2.21)	0.6
Post-traumatic	1.68 (0.55, 5.17)	0.4
Inflammatory	2.45 (0.68, 8.77)	0.2
Recurrent stricture vs. previously untreated stricture	1.50 (0.81, 2.79)	0.2
Optilume vs. Internal urethrotomy	0.63 (0.28, 1.42)	0.3

*CI* Confidence Interval,* HR* Hazard Ratio

## Discussion

The adoption of DVIU for the treatment of anterior urethral strictures results in recurrence rates of about 30–60% at 2 years [11, 14]. Formation of scar tissue is thought to result from incisional healing without modulation of the underlying scar formation biology [11]. Consequently, repeated DVIUs may lead to shorter intervals of recurrence and may compromise subsequent reconstructive options, highlighting the need for alternative procedures with more durable long-term outcomes [1, 3, 11]. Optilume has emerged as an alternative treatment strategy in this field, yet its advantage in comparison to existing treatments remains unclear [15]. This is the first study that directly compares ODCB dilatation and DVIU for the management of short anterior urethral strictures. The two treatment groups differed substantially regarding prior treatment history. While most patients undergoing DVIU did not have prior treatment (9%), the majority of patients treated with ODCB had undergone previous stricture interventions (78%), reflecting current real-world use of ODCB primarily as a salvage treatment after failed endoscopic management [1, 3]. A key consideration when interpreting our findings is the marked imbalance in treatment history between groups. ODCB was predominantly used after failed prior urethrotomy or dilatation, whereas DVIU was mainly performed as first-line treatment. Therefore, this study should not be interpreted as a direct comparison of equivalent treatment cohorts, but rather as an assessment of whether ODCB can achieve comparable outcomes despite being applied in a more challenging setting. Altogether, the findings of our study suggest that ODCB dilatation offers advantages over standard DVIU in terms of symptomatic improvement, reflected by a significantly greater reduction in IPSS at three months (12 points in the ODCB group vs. 4.5 points in the DVIU group), while demonstrating comparable short-term stricture-free survival despite being used in a substantially more pretreated and therefore more complex patient population [16]. Furthermore, a trend toward greater improvement in peak urinary flow was observed in the ODCB group (11.9 mL/s) compared to the DVIU group (7.4 mL/s), although this difference did not reach statistical significance.

Our study is relevant for several reasons. First, SFS was similar between Optilume™ DCB dilatation and DVIU. Although crude recurrence rates were numerically lower after ODCB (29%) in comparison to DVIU (63%), Kaplan–Meier analysis demonstrated similar estimated 12-month stricture-free survival between groups. This apparent discrepancy is explained by the significantly shorter follow-up in the Optilume™ cohort and the time-adjusted nature of survival analysis. This highlights that both strategies offer similar treatment success rates and can be effectively used in patients with anterior urethral strictures. However, this finding must be interpreted in the context of important baseline differences between groups. In our cohort, patients undergoing Optilume™ treatment had a substantially higher rate of prior stricture interventions, which might lead to poorer outcomes in the Optilume™ cohort. Moreover, the SFS rates in our study are lower than in certain single-arm Optilume™ studies, such as the ROBUST I and III trials [7, 8, 14]. These differences are likely reflected by differences in patient selection and study design. The comparable SFS between the treatment groups should therefore not be seen as equivalence of the procedures, but rather as a signal that ODCB might offset adverse baseline characteristics associated with poorer outcomes. In this regard, our cohort included many patients with more complex strictures in form of multiple prior interventions and longer strictures, both factors known to influence outcomes negatively [1]. Our study therefore reflects real-world usage patterns more accurate, as the ODCB is more often used after failure of DVIU [1, 3, 10]. 

Second, patients treated with ODCB experienced a more significant reduction in IPSS after 3 months compared to those undergoing DVIU, despite similar preoperative symptom severity. This suggests that ODCB may give patients superior symptomatic relief, which is a key feature of patient satisfaction and quality of life. This is consistent with results reported in prospective trials such as the ROBUST I and ROBUST III studies, which showed significant improvements in urinary symptoms following ODCB treatment [7, 8]. A plausible biological explanation is the combined mechanical dilatation and topical antiproliferative application of paclitaxel delivered into the scar tissue. Unlike DVIU, which relies on incision of the scar tissue alone, ODCB targets the underlying fibrotic process of scar tissue formation by inhibiting fibroblast proliferation [5, 12, 13]. This may lead to a reduced inflammatory response, which may reduce the formation of scar tissue, and therefore improve short-term functional outcomes and symptom relief, as reflected by the lower IPSS scores observed in our cohort.

Third, safety outcomes were comparable between the two treatment options. Both treatment options were associated with low rates of postoperative complications, with none reported as severe. This confirms that Optilume™ DCB may be safely used in routine clinical practice, especially in patients with many comorbidities and in more complex recurrent strictures. The similar duration of catheterization and low complication rate further support its suitability as an outpatient or short-stay procedure.

Previous studies have demonstrated high recurrence rates following DVIU, particularly with increasing follow-up duration and repeated interventions [11]. This led to guidelines not recommending repeated urethrotomy. Early prospective studies regarding ODCB, most notably the ROBUST trial series, have reported high stricture-free rates in selected patient populations, which led to its inclusion in EAU guideline recommendations for recurrent short bulbar strictures when urethroplasty is not an option [1, 7, 8, 15, 17, 18]. While EAU guidelines allow the use of ODCB in patients with recurrent short anterior strictures, our data suggest that ODCB may be an alternative early-line or even first-line option in selected patients.

This study is subject to some limitations. First and foremost, the retrospective design introduces inherent limitations that limit the extent to which the findings can be generalized. Second, patients treated with ODCB dilatations were more often treated for prior strictures, reflecting current real-world practice but limits accurate comparability between the two groups. In addition, follow-up duration was significantly different between DVIU with a median follow-up of 12 months and ODCB dilatations with a median follow-up of 9.5 months. Furthermore, outcome assessment was based on symptomatic recurrence, rather than endoscopic surveillance, which may lead to an underestimation of asymptomatic restenosis. Moreover, although multicenter, the cohort size limits the power to identify independent predictors of recurrence. Finally, the lack of long-term follow-up does not allow conclusions regarding durability, but the observed outcomes support the growing role of Optilume™ DCB as an at least intermediary option between repeated endoscopic incision and open urethral reconstruction, if not primary option for short anterior urethral strictures [9]. 

## Conclusion

This study presents the first comparative analysis between ODCB dilatation and DVIU. ODCB dilatation shows comparable outcomes with DVIU regarding SFS, and superior short-term symptomatic improvement, despite being used more often after prior treatment. Further prospective investigations with longer follow-up and larger and more comparable cohorts regarding prior interventions could help to redefine the current treatment algorithm for anterior urethral strictures.

**Fig. 1:**
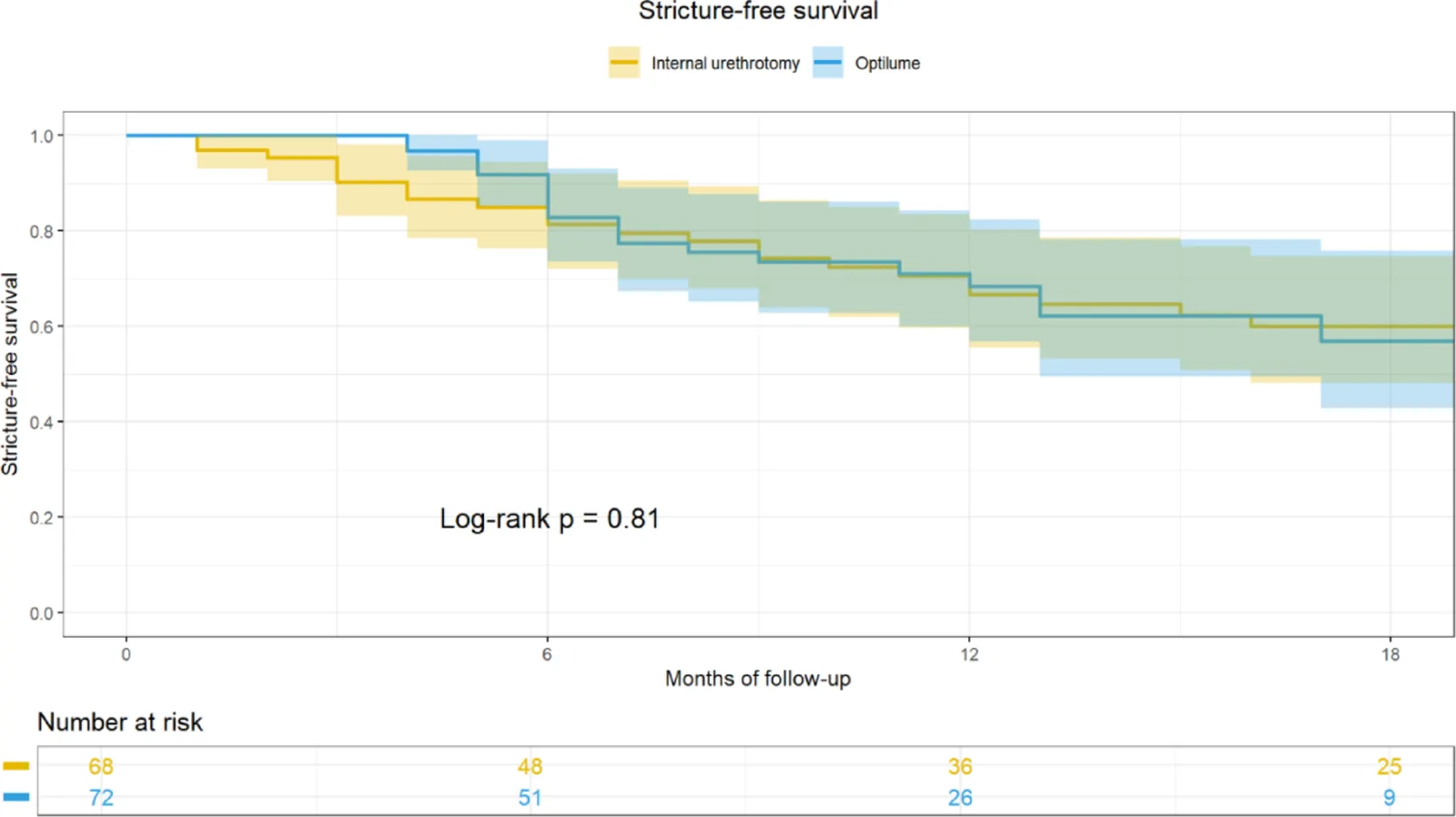
Kaplan Meier curves on stricture-free rate in 140 patients treated with internal urethrotomy or Optilume for anterior urethral strictures. Variables are expressed as median and interquartile range

## Data Availability

No datasets were generated or analysed during the current study.
